# Barrier Products for Topical Delivery—Insight into Efficacy Testing and Barrier-Boosting Compounds

**DOI:** 10.3390/pharmaceutics17111361

**Published:** 2025-10-22

**Authors:** Zofia Helena Bagińska, Emilia Szymańska

**Affiliations:** Department of Pharmaceutical Technology, Medical University of Bialystok, Mickiewicza 2c, 15-222 Bialystok, Poland; zofia.baginska@sd.umb.edu.pl

**Keywords:** barrier efficacy, topical product, skin protectant, clinical study, TEWL, permeability test, percutaneous absorption

## Abstract

The barrier effect refers to the ability of a topical product to protect the tissue against environmental factors, restore epidermal barrier function, or alleviate complications upon chemoradiation therapy. The market of barrier products for topical delivery is experiencing increased growth, and novel barrier-boosting compounds are being developed. However, only scarce reports and limited evidence justify their barrier efficacy. This may be due to the lack of a standardized, robust method for testing the protective effect of topicals. The paper reviews the recent advances in clinical and experimental techniques on the barrier efficacy of topical products, emphasizing those with the highest standardization potential. The principles and applications of each approach are specified, and the factors affecting the research outcome are highlighted. For predictive results, it is advised to mix at least two methods that differ in the mode of barrier efficacy testing. Combining quantitative TEWL and qualitative permeability testing not only balances out the limitations of each technique but also helps build high-quality evidence on the barrier efficacy. This review also summarizes the novel barrier-boosting ingredients and recent topical formulation strategies for enhancing product barrier efficacy and restoring the epithelial barrier function.

## 1. Introduction

The skin and mucous membranes are the key organs that serve as first-line barriers against external factors. In recent years, there has been an increase in the number of topical products that improve the epithelial barrier function, protect from allergens, irritants, or harmful agents, delay progression, and alleviate complications during cancer treatment. The barrier effect results from the action of components present in the preparation. The peptides, growth factors, or natural extracts (e.g., Cannabis sativa extract) accelerate the upregulation of keratinocyte differentiation, lipid and hyaluronic acid production, or expression of aquaporins 3 [1,2,3,4]. Bacterial metabolites and yeast extracts strengthen the epithelial integrity by, e.g., promoting the production of ceramides and filaggrin [5,6]. The nonphysiological lipids (petrolatum, paraffin, silicone) act as a mechanical barrier, occlude the skin, and prevent water loss [7,8,9]. In contrast, biolipid components (fatty acids, ceramides, cholesterol) stimulate the production of physiological lipids in the stratum corneum [7,10]. The other functional ingredients (e.g., Aloe vera leaf extract) improve skin hydration, exert a shielding effect (e.g., calamine, tannins), or participate in skin repair and remodeling (e.g., hyaluronic acid) [11,12,13]. Despite a gradual increase in barrier compounds and products, only limited scientific data support their barrier efficacy. This may be due to the lack of a consistent, robust technique to determine their protective effect.

**Figure 1:**
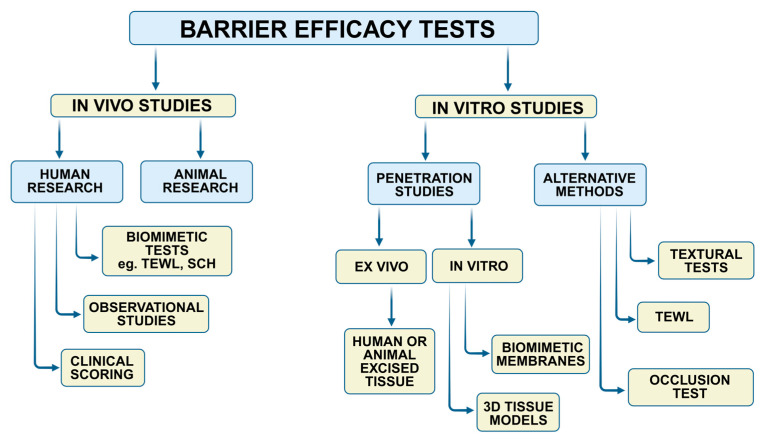
In vitro and in vivo strategies for testing the protective properties of topical products (Note: TEWL—Transepidermal Water Loss, SCH—Stratum Corneum Hydration, 3D—Three-dimensional).

According to scientific literature, numerous clinical and experimental studies have been developed to test the barrier efficacy of topicals (Figure 1).

These methods differ in terms of category of irritant agent, barrier model, dosing frequency, and quantification analysis [14]. Consequently, attained data is difficult to compare among studies, and barrier efficacy often remains controversial regarding reflecting real exposure.

The present article briefly reviews the in vivo and in vitro methods available for determining the barrier efficacy of topicals. The precise focus is on techniques with the highest implementation potential. The principles and applications of each approach are specified, and factors affecting the research outcome are outlined. This review also highlights the recent strategies to improve epithelial barrier integrity and innovations in barrier compounds and formulations. The effect of product composition on the final barrier efficacy is also discussed.

## 2. Methodology

**Figure 2:**
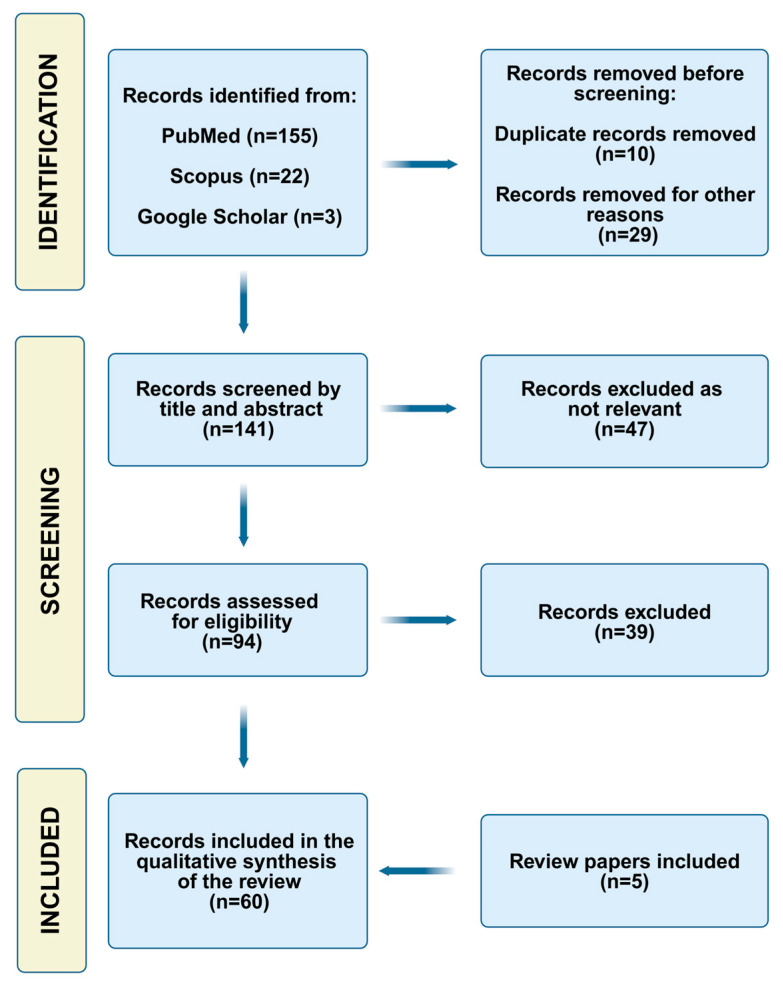
PRISMA chart of the search strategy.

A systematic literature search (Figure 2) was conducted according to PRISMA guidelines to identify relevant scientific studies published between 1995 and 2025. This enables us to answer the following questions: What is the current state of knowledge on the barrier efficacy testing of topical products? What in vitro and in vivo methods enable the assessment of the protective effect of topicals? Further research requests relate to the role of novel barrier ingredients and recent topical formulation strategies for enhancing product barrier efficacy, as well as understanding the factors affecting the research outcome in barrier testing.

The electronic databases PubMed, Scopus, and Google Scholar were searched for a combination of keywords such as: “barrier efficacy”, “barrier product”, “protective effect”, “barrier cream”, “barrier properties”, “barrier testing”, “skin protectant”, “barrier function”, “penetration”, “delivery”, “transport”, “permeation”, “diffusion cell”, “preparation”, “formulation”, “topical”, “cosmetic”, “medical device”, “emulsion”, “emollient”, “measurement”, “TEWL”, “performance”, “barrier-boosting”, “effectiveness”, “product composition”.

The article selection process was performed in two stages: prescreening titles and abstracts, followed by full-text evaluation. The potentially eligible articles were retrieved and assessed against the predefined inclusion and exclusion criteria. Inclusion criteria were clinical and experimental studies (in vivo, in vitro) on barrier efficacy testing, the studies aimed at evaluating the barrier effect of topicals, including medical devices, drug products, and skin care formulations, and the barrier effect of products intended for oral, vaginal, and skin delivery. Scientific papers aimed at invasive methods (including repeat insult patch or cumulative irritancy), those designed to examine skin barrier function without treatment, or to study factors and mechanisms responsible for skin impairment were excluded. Five additional review papers were retrieved through reviewing the references cited in the final stage of searching.

## 3. In Vivo Barrier Efficacy Assessment

In vivo barrier efficacy studies have been explored in animals and humans [15,16,17]. In the past, scientific papers mainly aimed at invasive methods such as the repeat insult patch, cumulative irritancy, or chamber scarification tests [18]. Currently, noninvasive, biomimetic methods are recommended, including transepidermal water loss (TEWL) and stratum corneum hydration (SCH) [15,19,20,21,22]. A summary of human experiments aimed at the protective efficacy of topical preparations is demonstrated in Table 1.

### 3.1. Studies in Human Volunteers

Human experiments are a valuable approach to determining the effectiveness of barrier preparations. They are based on biomimetic skin condition analysis or clinical scoring of disease symptoms. Double-blind, randomized, controlled studies are the gold standard as they provide consistent, unbiased data and help build the relationship between intervention and outcome.

### 3.2. Biomimetic Studies

#### 3.2.1. TEWL and SCH Measurements

**Figure 3:**
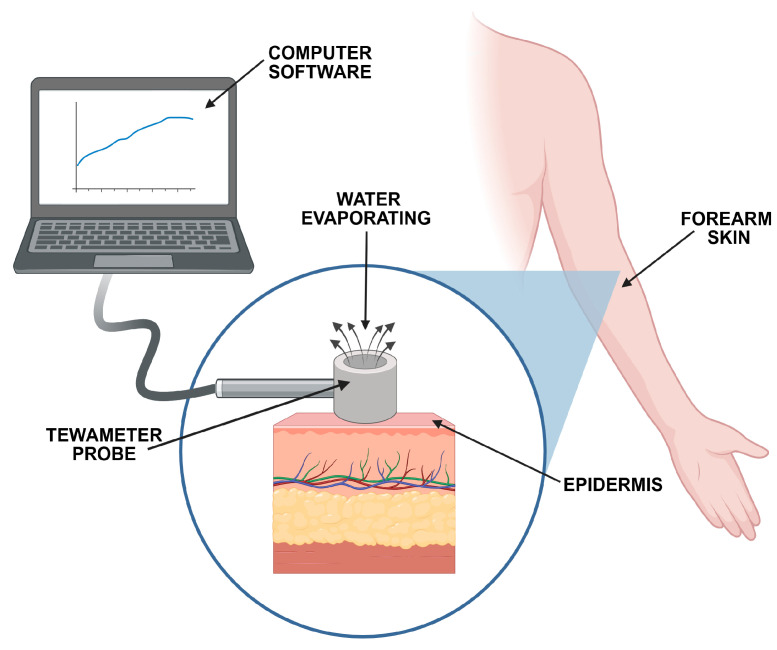
The scheme of TEWL measurement based on the open chamber evaporation method.

The TEWL technique measures the amount of water that diffuses across the stratum corneum [15,17,37] and can be used as an indirect tool to support the claims of the protective effect of topicals. For a healthy, intact skin barrier, the TEWL value is relatively low. In damaged skin, the water loss and TEWL values rise, usually proportionally to the degree of impairment. The commercially available measuring instruments can be divided into devices equipped with an open chamber (evaporimeter or tewameter) or a closed chamber (vapometer) (Figure 3). The use of all types is acceptable, though limited comparison studies between those instruments have been described.

The barrier properties can be additionally examined by stratum corneum hydration (SCH), a parameter based on electrical conductance [38]. While TEWL refers to water diffusion through the stratum corneum, the SCH represents the exact water content in the epidermis [39]. TEWL and SCH measurements can only produce reliable and reproducible data when performed under standardized conditions. European Group on Efficacy Measurement of Cosmetics and other Topical Products (EEMCO) identified critical variables affecting TEWL measurements (Table 2). They were implemented in EEMCO guidelines published in 2001 [40], and further supplemented by the Berardesca group [41].

Table 2 shows that a number of environmental or instrumental parameters, and broad intra- and interindividual variations, may impact TEWL analysis and lead to inconsistent results. Volunteers with healthy, normal-looking skin are recommended, though in certain cases, patients with impaired skin conditions can be included [19,23]. For instance, Danby group observed the reduced TEWL values after application of commercial skin care products in the form of a cream or emulgel, in volunteers with quiescent atopic dermatitis [23]. A single application test was performed on the volar forearms according to EEMCO guidelines [40]. Studies showed that test emulgel was associated with a prolonged skin hydration (up to 12 h) compared to the cream product. Overall, the authors concluded that neither formulation displayed sufficient barrier-strengthening properties to enhance skin barrier function.

In another study, Danby et al. examined the skin barrier-restoring effect of the skin care products with ceramides in male and female volunteers with eczema-prone skin [24]. Tests were performed at 21 ± 2 °C and 38–50% relative humidity after 20 min acclimatization to ambient conditions. Preparations were applied to the legs’ skin with an initial SCH value not lower than 35 AU. Tested products sustained clinically meaningful improvements in skin moisturization for up to 24 h compared to the reference emollients. The once-daily application of cream and lotion maintained the skin barrier effect and was comparable to the efficacy of reference products applied 2–4 times daily [24].

In TEWL and SCH measurements, the outcome can be recorded either after a single application [10,24,25] or upon long-term exposure [19,23]. The latter should be performed at comparable periods, after a defined product removal or cleansing procedure. Undried tissue shows a higher TEWL value due to gradual water evaporation from the skin [15]. According to Huygen et al. [42], TEWL is affected by daily routine activities. For instance, skin cleansing decreased TEWL for at least 15 min while increasing shortly after moderate to intense exercise.

Casiraghi et al. evaluated the protection efficacy with the TEWL parameter of cosmetic emulsions varied in water content. The measurements were conducted following the EEMCO guidelines in two variants: at a single point or pre-determined time intervals after application. The barrier effect was displayed as differences between control values established for petrolatum and those attained at each time point following topical application. The authors noticed that more lipophilic emulsions decreased water loss after just 15 min from application compared to control values, but the barrier effect was lower than that for petrolatum. Notably, the occlusive behavior of the most promising formulation with 55% water content was held over 5 h, which was comparable to the reference product [10]. In another study, Berndt et al. [19] tested the benefits of a barrier skin care product over 1 month under real workplace conditions according to EEMCO guidelines. A group of 50 hospital nurses with mild signs of skin irritation on their hands was treated either with commercial cream with aluminium chlorohydrate or its vehicle base (containing, e.g., liquid paraffin, dimethicone, and glycerin). The study displayed that the vehicle itself could enhance skin condition, and both tested products exerted comparable barrier efficacy. According to the authors, more than special ingredients, the regular, frequent product application is crucial for its protective efficacy.

Kubota et al. [28] introduced a barrier function index calculated based on TEWL and skin hydration measurements. The study aimed at evaluating the barrier functionality of skin care protectants with a complex of film-forming agents. The authors defined a test procedure but did not state whether they followed the current guidelines. Young, healthy volunteers were acclimatized at 20–22 °C, humidity level 40–60%, for 5 min before the experiment, and then the left forearm skin was covered with a cream and examined at pre-determined time intervals. The result attained after the first 5 min was used as the baseline value. Control studies with untreated skin or treated with cream vehicle without film-forming compounds were applied concomitantly. The authors underlined the potential of the barrier function index as a tool for evaluating the barrier efficacy of topicals. For the untreated control, increased TEWL values were observed, but no real changes in barrier function were noticed between skin areas covered with vehicle or tested cream [28].

Interesting studies were performed by Roure et al. [25], who evaluated the barrier efficacy of topical emollients in environmental simulated conditions. Before measurements, the volunteers were acclimated to testing conditions for 15 min at a temperature of 20 ± 2 °C and a relative humidity of 50 ± 5%. At the first stage, the emollient skin care product was applied to the forearms of 12 women for one hour before exposure to compressed air simulating the wind. In the chemical model, a patch with 1% sodium lauryl sulfate (SLS) was applied to cause moderate skin dehydration. SLS is an anionic surfactant frequently used to induce irritant contact dermatitis [43]. Pretreatment with a barrier cream containing oxozinc increased TEWL and reduced the dehydrating effect of SLS when compared to unprotected skin. However, it is difficult to state its real barrier effect as the protective function was evaluated solely against a ‘no treatment’ control [25]. In another study, Schliemann et al. induced contact dermatitis by application of sodium hydroxide or SLS solution on the participants’ back and examined differences in the barrier effect of commercial skin care products concerning the applied dose [26]. The volunteers were asked to acclimatize for at least 20 min to the controlled room conditions. Between analysis and applications, the test areas were covered by clothing, and the same investigator performed measurements. The protective efficacy of topicals was found to be dependent on the amount of product applied per surface area unit. High dose caused significantly higher hydration of skin irritated with SLS compared to unprotected control, while for several tested products, the low dose was less effective, indicating their insufficient protection.

Dykes et al. [27] tested the silicone-based creams (skin care products) in terms of resistance to the wash-off procedure. For this purpose, the forearm skin was covered with the product and subjected to a moisture challenge following three wash cycles. The authors identified the inclusion/exclusion criteria, volunteers’ baseline, and then specified the study protocol and wash-off procedure. Unprotected skin with no treatment was used as a control. Differences in TEWL represented alterations to the skin’s barrier function upon product application. Three out of five tested products demonstrated adequate moisture barrier protection and wash-off resistance, supporting their intended use as skin protectants from the effects of moisture exposure.

TEWL (and SCH) is a valuable tool, widely used as a non-invasive technique to measure barrier function. Despite all the benefits, TEWL and SCH are time-consuming tests and therefore unsuitable for screening studies. Both are relatively difficult to compare across various studies, as there is no defined ‘physiological’ TEWL or SCH value for healthy skin. TEWL values can be affected by environmental conditions, fluctuations in skin temperature, and initial water content. Numerous variables must therefore be initially checked and controlled for proper method design. EEMCO recommendations help set the testing conditions and distinguish critical factors affecting these parameters. To avoid the risk of misinterpretation, a relevant control (e.g., market product with confirmed barrier efficacy) together with control TEWL measurements at baseline and following topical application should be included in the testing procedure.

#### 3.2.2. Squamometry

Squamometry has been proposed as an alternative method for testing barrier products. It is a minimally invasive technique that measures the concentration of corneocytes of superficial skin layers removed by an adhesive disc [22]. The squamometric parameter C* value reflects the intensity of dye bound to collected cells [44]. Shimizu et al. demonstrated the barrier efficacy of a liquid containing tannic acid by D-Squame^®^ sampling. The product was applied on the forearm of healthy volunteers 30 min before patching with SLS solution as a model irritant [29]. The authors defined a skin barrier effect of tannic acid against the used surfactant.

### 3.3. Barrier Product Efficacy in Mucositis—Clinical Scoring

An important part of available clinical data concentrates on the efficacy of barrier products for mucositis treatment. Oral mucositis is a common complication during chemotherapy and radiation therapy. Conventional treatment includes ice chips, gastric tubes to improve nutritional condition, topical analgesics, antiseptics, and antimicrobial agents [45]. According to data, mucoadhesive delivery systems and coating agents are an effective alternative to delay progression or alleviate the mucositis symptoms [30,33].

For instance, Yin et al. evaluated the therapeutic effects of oral mouthwash (no information about composition was provided) in patients with radiation-induced mucositis [30]. Mouthwash-treated patients showed better pain control and delayed disease progression compared to the control group receiving the sodium bicarbonate solution [30].

In another study, patients classified for radiation therapy who received Mucosamin^®^ spray (a medical device containing sodium hyaluronate and amino acids) showed a substantial decrease in symptom severity compared to the control group treated with a combination of nystatin, diphenhydramine, magnesium hydroxide, and aluminum hydroxide [31]. Similarly, Cao et al. demonstrated that patients with chronic oral graft-versus-host disease receiving the oromucosal product (containing, e.g., polyvinylpyrrolidone and glycyrrhizinate salt) displayed less incidence of complications compared to the non-treated group [32]. Ala et al. evaluated sucralfate mouthwash (drug product) as a prevention strategy for 5-fluorouracil-induced oral mucositis [33]. The patients receiving sucralfate experienced delayed progression of mucositis and less intense pain vs. the placebo group. Soutome et al. examined the effect of oral liquid Episil^®^ (a medical device containing soy phosphatidylcholine and glycerol dioleate) on the pain management caused by radiation-induced mucositis in 15 patients with head and neck cancer [34]. The findings indicated comparable or inferior analgesic effects of the commercial formulation compared to dexamethasone ointment. In another clinical study, Episil^®^ was found effective in relieving pain and improving eating functions in 37 patients with hematologic malignancies [46]. A randomized, double-blind, placebo-controlled trial assessed the efficacy of Mugard^®^ (a medical device, carbomer-based hydrogel) as an intervention for chemoradiotherapy-induced mucositis. Mucoadhesive formulation used 4 times daily throughout the radiotherapy was more effective than bicarbonate saline rinse in alleviating symptoms and delaying mucositis progression [35].

Some systematic reviews analyzed the effectiveness of antiseptic mouthwashes in preventing the periodontal microbial biofilm formation [47,48]. According to Takenaka et al., a systematic use of preparations containing chlorhexidine gluconate or essential oils sufficiently protects from bacterial adhesion to oral tissues [48]. Ren et al. pointed out that short-term use of chlorhexidine salts prevents microbial colonization and reduces the occurrence of gingivitis in orthodontic patients. Authors noticed that mouthwashes containing natural plant extracts exerted barrier properties against pathogens, though their efficacy was lower than that of products with chlorhexidine [47].

### 3.4. Barrier Product Efficacy in Dermatitis—Clinical Scoring

Despite a wide range of emollients available on the market, a relatively limited number of studies have tested their barrier effectiveness. For instance, an open, randomized controlled trial evaluated the benefits of ‘emollient plus’ in patients with atopic dermatitis. Studies revealed that the tested product restored physical barrier function, maintained skin microbiome diversity, and reduced corticosteroid consumption compared to conventional emollient cream [36]. Fowler examined the effects of a commercial foam containing dimethicone in patients with chronic hand dermatitis [49]. Overall improvement in dermatitis symptoms was observed in 70% of the participants over 6 weeks of application. In addition, 16 out of 30 subjects reduced the use of topical corticosteroids. Another study by Fowler determined the degree of dermatitis improvement by barrier cream containing quaternium-18-bentonite [50]. Tested preparation reduced the disease severity and the need for topical corticosteroid application.

Rowe et al. summarized the clinical data evidence related to the efficacy of barrier products for treating nappy dermatitis [51]. According to the authors, applying ointments containing oxozinc and petrolatum provides sufficient protection and helps reduce the severity of symptoms in a mild, uninfected form of nappy dermatitis.

Despite clinical studies being the most reliable tool in determining product efficacy, several research papers point out the insufficient quality and lack of standardization within studies [52]. The main limitations are related to, e.g., short study period, omission of a real control group, and challenges with statistical analysis resulting from the small number of participants. In addition, strict inclusion criteria such as impaired skin condition can limit the findings to therapeutic, but not the protective properties of barrier preparations.

### 3.5. Animal Studies

Animal models for barrier efficacy testing include rodents (rats, mice), rabbits, and pigs, selected for their relevance to specific barriers like skin or mucosal epithelial tissue. Notably, according to ISO-10993-2 standards for the biological assessment of medical devices, studies on animal models should be reduced and replaced with in vitro techniques [53]. In response to European regulations, the use of animals in skin care products is no longer permitted. In fact, only a few scientific papers aimed at preclinical barrier efficacy have been published over the last two decades. Dachir et al. examined the efficacy of skin protectant Dermostyx^®^ (a glycerin solution with magnesium sulfate) in a pig model exposed to toxic agents (sulfur mustard, VX, ethyl parathion solution, acrolein) [54]. A single application before exposure to harmful agents significantly reduced the severity of skin lesions and alleviated systemic symptoms. Highly effective protection was attained, particularly against ethyl parathion and acrolein. The barrier properties remained effective for up to 12 h after application [54]. The paper presented by Chilcott et al. evaluated the efficacy of barrier formulation in reducing the percutaneous toxicity of warfare agent VX in a pig model [55]. Studies demonstrated that skin pretreatment with a product comprising polyperfluoromethylisopropyl ether (70%) and polytetrafluoroethylene (30%) substantially decreased skin contamination by VX, and eliminated mortality when compared to control (exposed to VX, no barrier cream) [55].

## 4. In Vitro Barrier Efficacy Testing

### 4.1. Permeability Studies

**Figure 4:**
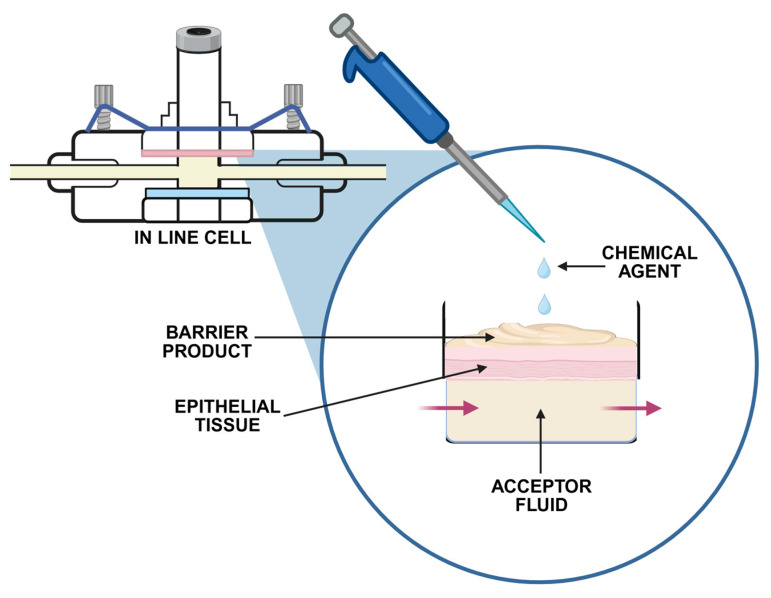
The scheme of the in vitro permeation study with an in-line diffusion cell.

In vitro studies are often faster and more reproducible and, therefore, considered a reliable screening tool for barrier candidates in topical formulation development. Among in vitro tests, permeability (penetration, percutaneous absorption) studies are the most commonly applied to examine the barrier efficacy of topical preparations (Table 3). This method involves measuring the passive diffusion of chemical agents across biological membranes (Figure 4).

**Figure 5:**
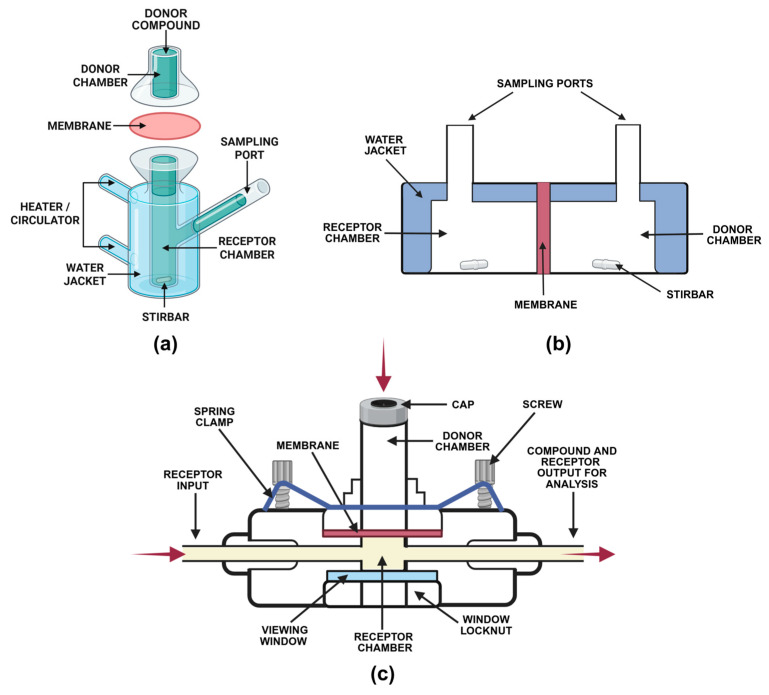
Types of diffusion cell systems used for in vitro permeation studies: (**a**) Franz diffusion cell; (**b**) side-by-side cell; (**c**) in-line cell.

The equipment for permeability studies consists of the donor chamber containing the chemical agent and the receptor chamber with fluid to collect the permeated fraction. Both are separated by a membrane, on top of which barrier preparation is placed [63,64]. Depending on the donor chamber, Franz cells, in-line and side-by-side chambers, can be distinguished (Figure 5).

According to guidelines on dermal absorption established by the Organization for Economic Cooperation and Development and the European Committee on Cosmetic Products and Non-Food Products intended for Consumers, it is a valuable tool for predicting topically delivered therapeutic agents’ absorption rate and efficacy [65,66,67]. One of the most frequent environmental and occupational skin damages is related to chronic exposure to mild irritants. As topical preparations create a physical layer between the epithelium and irritant factor, they reduce, delay, or temporarily block its penetration across the membranes. The level of protection is usually expressed as the percentage of absorbed fraction relative to the control, corresponding either to the untreated membrane (or tissue) or the membrane treated with the reference barrier product.

Penetration studies appear to be a helpful tool at the development stage of searching for the components with the highest barrier effect, or to compare the effectiveness of available topical products. According to Millerioux group [60], this test can also eliminate formulations that enhance the permeation of chemicals. However, until now, there has been no standardized procedure to evaluate the barrier efficacy of topicals with a permeability approach. Upon experimental design, several key parameters, including membrane type, acceptor fluid volume, chemical agent category, and exposure time, should be considered to understand the rules and correctly predict the barrier behavior.

#### 4.1.1. Factors Affecting Penetration Measurements

##### The Type of Membrane

Human [64] or animal tissue [68] and synthetic membranes [69] can be applied in penetration tests. Human samples can be donated from surgical biopsies, cosmetic surgeries, or cadavers. Among the animal tissues, the porcine epithelium is the most preferred as it has a high resemblance to human skin [70,71]. Both fresh and frozen tissues are acceptable. Freezing tissues immersed in saline solution at −20 °C is common, though storage at temperatures ≤ −80 °C is also practiced. Several studies confirmed that the freezing stage does not affect skin barrier integrity [72,73]. In contrast, Harrison et al. demonstrated that too harsh a temperature altered excised skin structure, indicating −20 °C as suitable for tissue storage [73]. Long-term conditioning (especially with acceptor fluid containing cosolvents or surfactants) should be avoided because of the risk of impaired tissue integrity over time [74].

Alternatively, synthetic membranes can be employed as a cost-effective and reproducible tool to examine barrier properties. Their use ensures data repeatability and does not require bioethical or ethical committee approvals [75,76]. Several types of membranes can be used, including cellulose, polycarbonate, silicone, and multilayered lipid-based membranes (e.g., StratM^®^ or PermeaPad^®^). It should be noted that the penetration pattern through artificial membranes may be affected, e.g., by interactions between their constituents and the chemical agent. Therefore, the synthetic membrane should be initially checked for compatibility with the tested product and chemical agent. Millerioux et al. showed that a silicone membrane could screen for effective barrier formulations against warfare agent XV exposure [56]. However, the authors emphasized that the relevant ranking of the most effective preparations requires further studies with tissue samples. In another study, Coderch et al. [57] displayed a comparable permeability pattern of four model drugs that differed in solubility through the polycarbonate membrane and the porcine sublingual mucosa. The experiment aimed to strengthen the oral mucosa’s barrier function with developed waterproof formulations. The artificial membrane showed much higher flux than the epithelial sample. Bignon et al. [58] examined the barrier efficacy of creams with cerium oxide (CeO_2_) nanoparticles against paraoxon exposure using human skin, silicone membranes, and Strat-M^®^. Both artificial membranes displayed substantially higher delay (up to 4 times) in the penetration rate of the chemical agent vs. human skin. Good correlation in penetration flux was observed between studies with StratM^®^ and human skin, but not with silicone membrane [58]. In contrast, the efficacy of the silicone barrier lasted up to 6 h, while longer effectiveness was observed in the Strat-M model [58]. Bassetto et al. proposed an in vitro approach to study the barrier efficacy of the medical devices by using cellulose membranes impregnated with a mixture of phospholipids, cholesterol, and n-octanol. The authors demonstrated that prepared membranes mimic gastrointestinal, buccal, and skin tissue and may be exploited as a feasible approach for barrier testing of topicals [59].

Overall, synthetic membranes can be considered predictive tools for screening studies. Importantly, the membrane integrity should be confirmed before measurements and at the end of the test. For animal or human samples, TEWL measurements can be performed, while for artificial membranes, a simple colorimetric assay, e.g., with methylene blue dye, can be used [59].

##### The Acceptor Solution

The amount of chemical agent that diffuses across the barrier product to reach tissue or membrane is evaluated by taking samples from the acceptor medium. Its composition and volume depend on the solubility of the chemical agent and its affinity to the membrane. In experiments with freshly excised tissue, a physiological solution, such as a cell culture medium, is recommended to maintain the physiological activity [77]. In the studies with stored, defrosted tissues, isotonic saline or buffered saline solution with pH 7.0–7.4 is adequate [15]. Non-physiological pH values are acceptable if the chemical agent is pH-dependent and displays varied ionization states. Notably, isotonic solutions (with values 280–330 mOsm/L according to [78]) are preferable to maintain tissue integrity. Strongly hypotonic or hypertonic media may induce swelling or cell shrinkage, respectively, and therefore should be used cautiously not to alter the penetration rate.

Sink conditions are a prerequisite to confirm that the permeated fraction of the chemical does not affect its further permeation. For this reason, the volume should be at least three times larger than required to obtain a saturated solution. If the agent has limited solubility, introducing solubility-enhancing agents (e.g., cosolvents or surfactants) is an approach to resolve the sink conditions. In this case, however, the additives may alter tissue integrity, specifically when the study is performed for an extended period. For instance, the presence of ethanol (especially at concentrations ≥10%) or polyethylene glycol with proven penetration enhancement properties is expected to facilitate flux, and in consequence, hinder barrier efficacy [74,79,80]. Therefore, large concentrations of these compounds should be avoided in barrier testing.

##### The Chemical Agent

Different substances, including warfare agents, surfactants, drugs, and dyes, can be applied in barrier efficacy testing as model irritant agents. Several factors must be determined when selecting a chemical, such as molecular weight, solubility, and partition coefficient. Those classified as ‘highly permeable’ should be chosen for penetration studies. At present, mild irritants are preferred, such as drugs (e.g., salicylic acid) [81], compounds used in medicine (in particular, caffeine) [57,64], or cosmetology (niacinamide) [82]. Irritants such as SLS, sodium hydroxide, and lactic acid (standards in the Repetitive Irritation Test) are rarely applied [83].

Regarding barrier efficacy testing, using warfare compounds is a valuable approach to simulate chemical environmental exposure [55,84]. For instance, Chilcott et al. [55] evaluated the protective efficacy of prototype cream with polyperfluoromethylisopropyl ether and polytetrafluoroethylene against warfare agent VX. Skin samples exposed to XV with no treatment served as control. The study design did not include any reference product. Short-term pretreatment of excised porcine skin with a tested formulation reduced the permeability of VX, demonstrating its potential as an alternative to protective garments. In another study, Millerioux et al. [60] determined the barrier efficacy of ingredients in emulsion composition against neurotoxic organophosphorus compounds. Research data showed that, e.g., the solubility of the chemical agent in the water or oil phase was critical as it could limit or enhance skin barrier function, respectively.

An alternative approach that helps distinguish the level of protection is to use dyes that differ in water/oil solubility. Treffel et al. [85] tested the effectiveness of barrier products upon exposure to dyes eosin, methyl violet, and oil red O, which varied in n-octanol/water partition coefficients (0.19, 29.8, and 165, respectively). Both hydrophilic and hydrophobic dyes penetrated the skin easily despite the presence of barrier creams. Data showed that the protective effect lasted for about half an hour. None of the tested products demonstrated absolute barrier efficacy except for petrolatum against eosin.

Recently, Magnano et al. examined the skin care products following exposure to nickel Ni nanoparticles [61] or Ni powder [62]. Two products were used in tests: the commercial cream Nik-L-Block^TM^ containing a chelating agent (pentetic acid) and a moisturizing basic cream Ceramol 311. Both creams reduced Ni accumulation in skin samples compared to control (not protected tissue), with pentetic acid-loaded cream being more effective. Interestingly, the permeated fraction of Ni was relatively high for skin samples pretreated with the tested creams compared to unprotected tissue, suggesting these formulations are relatively ineffective in long-term Ni nanoparticle exposure.

It should be highlighted, the chemical agent is present in the donor chamber as a solution with an infinite or finite dose. In contrast to penetration studies where both dosing regimens are acceptable [74,79,86], in testing barrier effects, an infinite dosing of chemicals should be considered to maintain their constant absorption rate and assure the steady-state flux [61,62].

#### 4.1.2. General Considerations for Barrier Testing with an In Vitro Penetration Study

The degree of protection is a variable, and penetration tests can discriminate the barrier strength of topical products. Penetration studies can produce reliable data only under defined and controlled conditions. Based on collected scientific papers, Table 4 summarizes the general recommendations for barrier efficacy testing with a penetration approach.

To reduce the risk of misinterpretation, a reference control, e.g., a commercial barrier product with a proven protective function, should be included. Regarding model irritants, in some cases, hydrophilic and lipophilic chemicals could give complementary information on the efficacy spectrum of barrier topicals towards various chemicals [60]. Combining two chemical agents that differ in hydrophilic/hydrophobic behavior is a particularly attractive approach to distinguish the penetration degree of chronic exposure to chemical irritants.

The pretreatment time with the irritant agent varies between the studies from 15 min [55] to several hours [10,15]. Analysis time and sampling schedule should distinguish the time point necessary to disrupt barrier efficacy and detect the presence of the chemical in the acceptor fluid. Casiraghi [10] suggested that a penetration test using a hydrophilic agent, caffeine, required approximately 3 h to display the permeation pattern.

### 4.2. Studies with 3D Tissue Models

Barrier efficacy can also be measured on the reconstructed three-dimensional (3D) human tissue cultures. There are biologically relevant models with high similarity in morphology and functionality to human tissues. In accordance with European guidelines, 3D tissue models are an attractive alternative to reduce the use of animals in preclinical testing. In contrast to artificial membranes, they are characterized by high biological variability and hence lower repeatability. These 3D models require special storage conditions and have a limited service life [87,88].

So far, only limited scientific data have involved 3D tissue models for barrier efficacy testing. Casiraghi et al. developed an experimental protocol for the quantification of the barrier properties of topicals with a reconstructed human epidermis model. The tested product was spread over the epidermis with confirmed integrity and left for 2 h at ambient temperature. The culture inserts were then transferred to a plate filled with saline fluid, and a caffeine solution (1% *w*/*v*, 0.1 mL) was applied to the surface of the tissue model. The receptor fluid was analyzed for caffeine content at a predetermined time. Control studies with untreated tissue and tissue treated with petrolatum as a reference barrier product were additionally performed. Tested creams varied in caffeine content after 3 h of incubation time. The barrier effect of petrolatum was confirmed, as no caffeine was present in the acceptor medium throughout the study. Some formulations showed a high permeated amount, not significantly different from those obtained in untreated tissue. Overall, the authors demonstrated that the 3D skin model was a fast and sensitive approach, providing results on the most effective barrier formulation within 1–3 h from the product application [10].

### 4.3. Alternative In Vitro Techniques for Barrier Efficacy Testing

Other in vitro alternatives are described in the literature besides the penetration studies. For instance, Kulawik-Pióro et al. investigated relationships between rheological and textural properties and barrier efficacy. Three commercial skin care products were selected for studies: preparations A and C (containing water, glycerin, and white wax or paraffin, respectively) and a more complex formulation B (comprising, e.g., emollients, xanthan gum, urea, panthenol, lactic acid, and allantoin). The method analyzed the consistency, spreadability, and structure recovery upon application [89]. The authors indicated that poor spreadability may decrease the quality of the protective layer. Formulation B (without paraffin or white wax) was more resistant to mechanical forces than the other analyzed products, and therefore was selected as the most barrier preparation.

Modified variants of corneosurfametry and corneoxenometry were introduced as ex vivo bioassays to predict and compare the protection efficacy of marketed skin protective products against model skin irritants [90]. For this purpose, skin surface was collected from 15 healthy volunteers by tape-stripping and exposed to model surfactant or organic solvent. Tap water served as a negative control. Data were expressed as a percentage of protection relative to the controls corresponding to water and each single applied irritant. The level of protection against irritant agents varied considerably among the tested formulations. The presented approach stressed the inadequate efficacy of several marketed products in contrast with claimed properties.

Commonly used in humans, TEWL measurements can also be applied for in vitro testing with tissue samples. For instance, Schoenfelder et al. [15] investigated the effect of different emulsifiers on the barrier integrity of porcine skin. The authors noticed profound changes in TEWL values regarding the tested barrier agent, incubation time, and study conditions, highlighting the importance of introducing additional control with untreated samples.

Trobos et al. [91] compared the barrier properties of non-resorbable polytetrafluoroethylene (PTFE) membranes, which were designed as a medical device for bone tissue regeneration. The barrier efficacy of two PTFE materials, which differed in microstructure pattern (expanded and dense type), was examined against the model pathogenic strain *Streptococcus oralis* (*S. oralis*). A profound reduction in bacterial biofilm formation in the presence of porous PTFE membranes was noticed. Despite both tested preparations being impermeable to *S. oralis*, the expanded type of PTFE membrane displayed substantially lower bacterial colonization after 48 h incubation [91].

Antonijević et al. [92] evaluated two dermatological products (registered as medical devices) with similar compositions (Isomol^®^ gel and Doublebase^®^ gel) by the occlusion test. The occlusion effect was calculated by the decrease in cumulative evaporative weight loss of the excised tissue upon 48 h application. Eventually, the authors distinguished the level of protection efficacy between products, but did not set up any control.

## 5. Novel Barrier-Boosting Compounds

Several novel ingredients with barrier-boosting properties have been developed recently. They display different levels of barrier efficacy depending on the intended use: protect from irritants or allergens, offer microbial resistance, promote healing, or accelerate regeneration. The active substances directly interact with the skin components. They may either target inflammation (e.g., peptides, tannins), accelerate the keratinocyte proliferation and differentiation (e.g., growth factors, natural extracts), enhance hyaluronic acid (e.g., protopanaxatriol) and lipid production (e.g., apigenin), or stimulate the expression of aquaporins-3 (e.g., retinoic acid derivatives). They can also bind and neutralize transition metals responsible for skin damage (e.g., chelating agents) or prevent free radical production and lipid peroxidation (e.g., resveratrol). Table 5 summarizes the active ingredients with epithelial barrier boosting activity.

At present, barrier products containing functional endocannabinoids are acquiring greater importance. Phospholipids like N-palmitoylethanolamine and N-acetylethanolamine, belonging to the functional endocannabinoid system, regulate the differentiation of epithelial cells, and thus can play an active protective role in skin barrier function [93,94]. It has also been demonstrated that the endocannabinoids inhibit inflammation and prevent skin damage from exposure to ultraviolet light [95]. According to a recent study performed by the Zonari group [96], the OS-01 peptide holds promise in restoring skin barrier function as it was found to decrease the levels of proinflammatory cytokines and reduce inflammatory response.

Wagemaker et al. [97] demonstrated that topical formulation with resveratrol reduces reactive oxygen species, inhibits inflammatory responses, and enhances skin barrier function. Recently, a new generation of microbial barrier products has gained interest in rebalancing the skin microbiome. These products are characterized by active ingredients influencing the skin microbiome, such as riboflavins from oat plantlet extracts or nonpathogenic bacteria. For instance, Zelenkova et al. examined the efficacy of a commercial product containing a lysate of *Vitreoscilla filiformis* for reducing inflammation and decreasing *S. aureus* biofilm formation in patients with atopic dermatitis [36]. Upon 28-day treatment, the tested formulation restored barrier function and reduced corticosteroid consumption. In another study, Lactobacillus strain LP51 derived from a healthy vaginal microbiome exhibited anti-inflammatory and antioxidant properties and demonstrated benefits in improving skin hydration [5].

Regarding barrier efficacy, novel delivery systems in the form of micro- or nanoparticulate carriers have also been evaluated. For instance, Bignon et al. examined the protection properties of six formulations varied in the amount of CeO_2_ nanoparticles covalently grafted to methacrylic acid, 2,2,2-trifluoroethyl methacrylate (HASE-F) in a penetration test with paraoxon (POX) [58]. The authors demonstrated that grafting CeO_2_ nanoparticles to HASE-F polymer reduced POX diffusion compared to the unprotected control. The skin pretreatment with cream containing 13% CeO_2_-HASE-F composition was the most effective against the toxic agent. Coderch et al. examined differences in protective properties between hydrophobic, hydrophilic, and liposomal formulations (Table 3) using porcine sublingual mucosa or polycarbonate membrane. The tested preparations were applied to the membrane surface and then exposed to chemical agents from different biopharmaceutic classification system (BCS) groups. Among the tested preparations, the liposomal formulation displayed the greatest barrier properties as it reduced the permeability of caffeine, ibuprofen, and dexamethasone by approximately 80% [57].

**Table 5:** Examples of active substances with barrier-boosting activity present in topical protectants.

Ingredient	Mode of Action	Outcome	Reference
Aloe vera extract	A hydrating, protective, and soothing ingredient with adhesive properties as well as film-forming ability	Creating a protective film, limiting the penetration of caffeine	Bassetto et al. [59]
Apigenin	Enhanced filaggrin expression and lamellar body production; upregulation of lipid synthetic enzymes	Improved skin barrier recovery	Hou et al. [4]
Cannabidiol	Enhanced expression of cytoprotective enzyme HMOX1 in keratinocytes; antioxidant, anti-inflammatory, and anti-apoptotic properties	Improved skin barrier recovery	Casares et al. [2]
Endocannabinoids (e.g., palmitoylethanolamide)	Anti-inflammatory effect; Stimulating the keratinocyte proliferation and differentiation;	Improved skin barrier recovery, increased epithelial hydration	Yuan et al. [93], Madnani et al. [94] McCormick et al. [95]
Enoxolone	Inhibition of endogenous hyaluronidase activity; increase in epidermal ceramide production	Enhanced skin hydration; improved integrity of intercellular cement	Zeichner et al. [98]
Pentetic acid	Chelating agent	Blocking the dermal accumulation of nickel	Magnano et al. [61,62]
Protopanaxatriol	Enhanced expression of transglutaminase, claudin, occludin, and filaggrin; stimulation of hyaluronic acid production; upregulation of Src/AKT/NF-κB signaling	Improved skin barrier recovery, increased epithelial hydration	Lee et al. [99]
Resveratrol	Antioxidant and anti-inflammatory activity; inhibition of free radical production and lipid peroxidation	Improved skin barrier integrity	Wagemaker et al. [97]
Microbiome therapeutic Lactobacillus strain LP51	Modulating the skin microbiome composition; anti-inflammatory, antioxidant properties; increased the transcription of genes: HAS3, FLG, IVL, and LOR	Enhanced skin hydration; improved the skin barrier integrity; xerosis treatment	Kim et al. [5]
Tannic acid	Re-epithelialization enhancement; forming an impermeable layer with skin proteins	Skin barrier restoration: a physical barrier against irritants	Nakamura et al. [100]

Note: HMOX1—heme oxygenase-1; Src/AKT/NF-κB—proto-oncogene tyrosine protein kinase/protein kinase B/nuclear factor kappa-light-chain-enhancer of activated B cells; HAS3—hyaluronan synthase 3; FLG—filaggrin; IVL—involucrin; and LOR—loricrin.

## 6. The Impact of Product Composition on Barrier Efficacy

The vehicle composition (base) is vital and can be as effective as the active agent in skin protection. Most barrier products, with proven protective properties, contain non-physiological lipophilic components, such as petrolatum, silicone, and paraffin. These ingredients are, in fact, frequently applied as positive control in barrier testing [83,85]. However, little scientific data compares their efficacy and indicates which lipophilic ingredient is more effective. Kurpiewska et al. evaluated the barrier creams containing beeswax or ozokerite as the main ingredients responsible for the occlusive effect. The researchers examined the permeability rate of SLS, acid, and alkaline solutions through cellulose membranes covered with the tested preparations. Studies showed that preparation containing beeswax exhibited higher barrier properties against irritant agents [101]. In a randomized, double-blind study, Martini et al. compared the efficacy of a topical cream and its vehicle on foot xerosis in diabetic patients upon 28-day treatment [102]. The tested product Dexeryl^®^ (a medical device with polydimethylcyclosiloxane and silicone) significantly enhanced the overall skin score, hydration index, and skin roughness compared to the vehicle. In turn, the scientific data published by Roure et al. showed no significant differences between the protective cream (with aluminum chlorohydrate) and its vehicle [19]. Incorporating physiological lipid components (e.g., cholesterol, fatty acids, ceramides) is now considered more beneficial for restoring skin barrier function, as they easily penetrate the epidermis, stimulating the production of intercellular lipids [103].

Importantly, inactive additives present alongside lipophilic ingredients may affect their protective efficacy. The more complex the composition is, the more the barrier effect may be impaired. Millerioux group [56] demonstrated that topical formulations could significantly change the tissue surface energy and barrier efficacy either through direct interactions with the epithelium (e.g., emulsions) or by forming an occlusive layer on the skin surface (e.g., perfluorinated-based formulations). Specific emollients and emulsifiers can sometimes interact with skin components and impact skin barrier function. According to Brunina [104], an emollient component in a topical product may exert a dual effect depending on the skin type, e.g., increased TEWL in healthy skin but reduced in damaged tissue. In particular, synthetic emollients/emulsifiers raised concerns about their barrier properties as they may impair skin condition over time. In addition, the presence of penetration enhancers, e.g., polyethylene glycol or unsaturated fatty acids, may diminish the efficacy of lipophilic compounds. Schoenfelder et al. investigated the effect of surfactants (polyethylene glycol (PEG)-based ethers, sorbitan esters, lecithin, and cholesterol) on the integrity of excised porcine skin. Among the tested substances, PEG-20 cetyl ether and PEG-20 stearyl ether disrupt the skin barrier comparably to SLS solution [15]. Reuter et al. elucidated the correlation between ceramide content and the skin barrier function when exposed to different pharmaceutically used emulsifiers. Among the tested substances, nonionic surfactants displayed no significant effect on the skin barrier [105].

In a cream formulation, the water content may affect the final protective efficacy. For instance, Casiraghi et al. evaluated topical emollients varied in water content (20–70%, *w*/*w*). They found that the formulation containing 55% of water was the most occlusive, while that with 70% had substantially impaired barrier efficacy [10]. In contrast, Treffel et al. demonstrated that neither the water content nor the formulation consistency impacted the protection efficacy [85].

Several studies point toward using hydrophilic polymers or hydrophilic (e.g., hyaluronate, polyvinylpyrrolidone, carbomer) or hydrophobic (e.g., perfluorinated) to facilitate barrier efficacy. Studies performed by Millerioux et al. revealed that a formulation containing hydrophilic polymers was more effective in skin protection against the nerve agent VX than a perfluorinated-based preparation [56]. Another study performed by Millerioux, however, demonstrated that perfluorinated compound-based cream displayed higher barrier efficacy against organophosphorus agents when compared to, e.g., water-in-oil emulsion (with polyvinylpyrrolidone, polyquaternium 51, and cetyl dimeticone copolyol) [60]. Allison et al. showed that carbomer-based oral medical device Mugard^®^ alleviated pain and delayed the progression of oral mucositis [35]. Bassetto et al. demonstrated barrier efficacy of Aphthae^®^ gel, a medical device that contains, e.g., xyloglucan, polyvinylpyrrolidone, and aloe vera extract. Studies showed reduced caffeine permeability by about 60% compared to an unprotected membrane. Notably, the tested preparation displayed about 30% lower efficacy when compared to petrolatum [59]. Another study demonstrated that sucralfate mouthwash, by forming a polyanion hydrogel, could be an effective prevention strategy for 5-fluorouracil-induced mucositis [33].

Valuable research was performed by Schliemann et al., who assessed the effect of the applied dose on the efficacy of commercial skin care creams in preventing contact dermatitis [26]. Three products that differed in composition (Table 1) were used at a low 2 mg/cm^2^ or a high dose of 20 mg/cm^2^. The higher doses of the preparations provided significant protection against NaOH-induced and SLS-induced irritation. In turn, the lower dose of two out of three products did not protect against SLS-induced irritation [26].

## 7. Future Perspectives and Application Prospects in the Barrier Testing Approach

The market of topical protectants is experiencing increased growth, but these products are not usually tested for barrier quality. Thus, their efficacy is controversial and not easy to prove from a regulatory point of view. There is a need for standardized direction guidance to test the protective efficacy of topical products that prove the intended use of market products and help at the development stage.

Given the above, it is clear that there is still no universal approach for efficacy testing of topicals. Each presented method has its advantages and drawbacks, and requires an entirely different setup. When carried out solely, the leading techniques require careful control and may result in invalid findings. For instance, the application of TEWL as the only measurement should be considered with caution, e.g., for simple-driven testing of waterproof skin care products. To provide high-quality evidence, a mixed-method approach should therefore be considered to merge the strengths and overcome the drawbacks of each method. Among the presented, a combination of qualitative TEWL measurements with quantitative permeability testing appears the most promising for effective barrier testing of topical products. Some scientific papers observed a correlation between TEWL and skin permeability studies [81,106,107], though several studies did not find any relationship between them [108,109].

Yoo et al. [106] examined the relationship between the percutaneous penetration of fluorescein sodium salt and TEWL in volunteers before and 5 days after skin damage by SDS treatment. Participants applied a barrier skin care cream on a damaged area twice daily for 5 days. The penetration rate was assessed using the tape-stripping method and fluorescence microscopy. Studies demonstrated a linear relationship between TEWL and percutaneous absorption. According to the authors, both parameters represent the skin permeability but in opposite directions. TEWL refers to in–out permeability as it indicates the water vapor lost from the body, whereas the penetration rate, which shows the amount of agent that has entered the skin from the external environment, represents out–in permeability. In another study, Tsai et al. [107] explored the percutaneous absorption of compounds differing in lipophilicity and compared it with TEWL measurements in vivo. Acetone treatment was used on a hairless mouse model to impair the skin barrier. The mouse skin was then excised and placed in diffusion cells for the penetration studies. The skin barrier disruption by acetone and TEWL measurements correlated with the skin permeability of the hydrophilic (sucrose, caffeine) and lipophilic drugs (hydrocortisone). In turn, a weak correlation between TEWL and the skin absorption of highly lipophilic compounds (estradiol and progesterone) was noticed. The attained results suggested the need to apply additional TEWL analysis and introduce chemicals varied in lipophilicity to better predict alterations in skin permeability. Benfeldt et al. [81] aimed to find the relationship between TEWL and skin permeability of salicylic acid in healthy participants. Penetration rate and TEWL values were monitored in untreated skin, skin damaged with tape stripping, and treated with SDS or acetone. The penetration of the drug significantly correlated with the measurements of TEWL in all variants of damaged skin.

Alsamad et al. [108] evaluated the correlation between TEWL and the penetration of topically applied caffeine in healthy skin before and following a cleansing procedure. In the in vivo study, the forearm skin of 9 healthy volunteers was challenged with a mild cleanser product (or water as a control) under occlusion for 3 h and checked for TEWL and the penetration rate of the model irritant by confocal Raman spectroscopy. A weak correlation was observed between TEWL values and the penetration flux of caffeine, suggesting that TEWL measurements are insufficiently sensitive to differentiate changes in skin hydration following the cleansing procedure. Thus, according to the authors, the TEWL parameter could be recommended only to discriminate large variations in skin barrier function, e.g., between healthy and impaired skin. Chilcott investigated the relationship between TEWL and excised skin permeability to validate the hypothesis that TEWL values reflect the real skin barrier function [109]. For this purpose, human and porcine epidermis samples were placed in diffusion cells and left to equilibrate for at least 24 h before TEWL and permeability measurements with tritiated water and sulfur mustard as model hydrophilic and lipophilic agents, respectively. The study revealed that basal TEWL values did not correlate with baseline skin permeability to water or sulfur mustard. Similarly, TEWL measurements failed to detect damaged tissue in vitro. These scientific data indicated that under certain experimental conditions, elevated TEWL rates should not be attributed to an alteration of skin barrier function in vitro.

It should be noted that artificial intelligence (AI)-driven predictive models have been recently introduced into dermatology and the cosmetic field to support the skin barrier efficacy assessment. For instance, to speed up the traditional TEWL approach, Koseki et al. [110] proposed an AI algorithm that uses skin images with topological data analysis to forecast barrier function. The authors demonstrated the strong correlation between structural characteristics and TEWL values, which gives the prospect of broader applicability of AI tools in development studies on topical protectants. AI-driven predictive modeling is expected to reshape the market of topical products (including barrier formulations) and boost research and development strategy in the near future.

Apart from the type of applied technique, the relevant control studies are critical to build valid evidence on the barrier efficacy. The use of a simple petrolatum with proven barrier effect (or a relevant bench market barrier product) as a reference should be included in the testing procedure. To avoid the risk of misinterpretation, the real effect needs to be presented as differences between the control and the tested product.

## Figures and Tables

**Table 1:** Examples of human experiments aimed at testing the barrier efficacy of topical products.

Method of Barrier Efficacy	Participant /Disease	Control Group	Type of Barrier Product	Reference
TEWL and SCH test	Volunteers with quiescent atopic dermatitis	No treatment, no skin protection	Skin care emollient cream (petrolatum, paraffin, silicone polymer) Commercial emollient gel (isopropyl myristate, paraffin, carbomer)	Danby et al. [23]
SCH test	Volunteers with eczema-prone skin	Reference emollient creams (skin care products)	Skin care cream and lotion containing ceramides (1, 3, and 6-II), cholesterol, and triglycerides	Danby et al. [24]
TEWL test	Healthy volunteers	Petrolatum ointment	Skin care creams differed in water content (20–70%, *w*/*w*)	Casiraghi et al. [10]
TEWL test	Healthy volunteers	No treatment, no skin protection	Skin care emollient with glycerin and sodium glycine	Roure et al. [25]
TEWL and SCH tests	Healthy participants with induced contact dermatitis	No treatment, no skin protection	Cream A (aluminium chlorohydrate, paraffin, urea, petrolatum, beeswax, cholesterol, zinc stearate, lanolin) Cream B (petrolatum, paraffin, glycerin, cera alba, zinc stearate) Cream C (kaolin, paraffin, petrolatum, oxozinc)	Schliemann et al. [26]
Resistance to wash-off, SCH tests	Healthy volunteers	No treatment, no skin protection	Commercial skin care silicone-based products	Dykes et al. [27]
TEWL and skin hydration tests	Healthy volunteers	No treatment, no skin protection	Formulated skin care creams (glycerin glyceryl isostearate, isopropyl myristate, squalene, retinol palmitate, ascorbyl tetraisopalmitate, stearic acid, simethicone) with or without film-forming agents (poly-perfluoromethyl isopropyl ether, silsesquioxane, myristoyl pullulan)	Kubota et al. [28]
Squamometric analysis	Healthy volunteers	No treatment, no skin protection	Tannic acid solution	Shimizu et al. [29]
Clinical scoring	Oncologic patients with radiation-induced mucositis	Sodium bicarbonate solution	Mouthwash (no information about composition)	Yin et al. [30]
Clinical scoring	Oncologic patients with radiotherapy-induced mucositis	Combination of nystatin, diphenhydramine, magnesium, and aluminum hydroxide	Commercial oral spray (medical device, sodium hyaluronate, glycine, L-leucine, L-lysine)	Nasrollahi et al. [31]
Clinical scoring, salivary flow	Patients with chronic oral graft-versus-host disease	No treatment	Commercial oromucosal preparation (medical device containing polyvinylpyrrolidone, trisodium glycyrrhizinate)	Cao et al. [32]
Clinical scoring	Oncologic patients with chemotherapy-induced mucositis	No treatment	Mouthwash with sucralfate (drug product)	Ala et al. [33]
Clinical scoring	Oncologic patients with radiation-induced mucositis	Dexamethasone cream	Commercial oral liquid (medical device comprising soy phosphatidylcholine and glycerol dioleate)	Soutome et al. [34]
Clinical scoring	Oncologic patients with chemotherapy-induced mucositis	Saline solution	Commercial oral carbomer-based hydrogel (medical device)	Allison et al. [35]
Clinical scoring	Patients with atopic dermatitis	Conventional emollient cream (no information about composition)	Commercial skin care product with Aqua posae filiformis, canola oil, niacinamide	Zeleknova et al. [36]

Note: TEWL—Transepidermal Water Loss; SCH—Stratum Corneum Hydration.

**Table 2:** EEMCO recommendations on TEWL assessment concerning selected test variables [40,41].

Volunteer-Linked Variables	Recommendations
Sex, age, race	Homogeneous group preferable Defined inclusion and exclusion criteria
Anatomical side	Volar forearms recommended Other sides are acceptable upon justification Forehead, palm, wrist should be avoided
Skin surface temperature and sweating	At least 15–30 min acclimatization period in a temperature- and relative humidity-controlled room Measured site remains uncovered before the test
Skin damage/disease	Skin damage procedure (tape stripping, acetone, or SDS treatment) has to be specified Relevant controls (e.g., on healthy skin spots from the same anatomical site) have to be included
Daily routine	Coffee intake should be avoided before measurements Physical activity limited to a minimum
Circadian rhythm	Measurement at the same time of the day
Product removal procedure	The effect of the procedure on TEWL should be preliminarily checked
Skin cleansing	The effect of the procedure on TEWL variations should be investigated
Environmental variables	
Temperature and humidity	Temperature and humidity conditions controlled Preferable temperature 20–22 °C, relative humidity < 60% The hot summer of freezing winter days should be avoided unless the study aims otherwise
Light source	Direct and close light sources should be avoided
Air circulation	Air circulation should be limited and monitored
Single experiment	Seasonal variations should be avoided If possible, performed by the same operator
Repeated or long-term experiments	Measurements at the comparable time periods (e.g., the same hour per day, defined time period after product removal or after cleansing procedure) Medical treatment and skin care products disturbing TEWL values should be avoided thorough experiment setup
Instrumental variables	
Measuring probe	Applied perpendicularly to the skin surface with a constant, light pressure Avoid touching before and during the test The probe should be warmed up to test an individual’s skin temperature
Analysis time	As short as possible to avoid occlusion Zero value should be displayed before measurement

Note: EEMCO—European Group on Efficacy Measurement of Cosmetics and other Topical Products; TEWL—Transepidermal Water Loss.

**Table 3:** Examples of in vitro penetration studies aimed to assess the barrier efficacy of topical products.

Membrane Model	Type of Irritant Agent	Control	Type of Barrier Product	Reference
Human abdominal skin, porcine ear skin, silicone membrane	Warfare agent XV	Unprotected membranes	Non-proprietary skin care cream with perfluorinated compounds; Commercial emulsion o/w with silicone, perfluorinated polymers, polyvinylpyrrolidone, glyceryl and PEG-100 stearate; Skin care emulsion w/o with polyvinylpyrrolidone, polyquaternium 51, and cetyl dimeticone copolyol	Millerioux et al. [56]
Porcine sublingual epithelium, polycarbonate membrane	Caffeine, ibuprofen, dexamethasone, ivermectin	Unprotected membranes	Hydrophobic skin care formulations (based on petrolatum, lecithin, isopropyl myristate, or medium chain triglycerides); Hydrophilic skin care formulations (with chitosan, CMC, poloxamer, alginate sodium, or hyaluronate sodium); Liposomal formulations (with ceramides-3, -6, cholesterol, or phosphatidylcholine)	Coderch et al. [57]
Human skin, Strat-M, silicone membrane	Paraoxon	Unprotected membranes	Non-proprietary cream formulations with cerium oxide nanoparticles grafted to methacrylic acid, 2,2,2-trifluoroethyl methacrylate	Bignon et al. [58]
Cellulose membrane impregnated with phospholipids, cholesterol, and n-octanol	Caffeine, acyclovir	Unprotected membrane (negative control), membrane covered with petrolatum (positive control)	Oral hydrogel with xyloglucan, aloe vera extract, glycerol, and polyvinylpyrrolidone (medical device)	Bassetto et al. [59]
Porcine ear skin, silicone membrane	Paraoxon	Unprotected membranes	Non-proprietary cream with perfluorinated compounds (medical device); Emulsion o/w with silicone, perfluorinated polymers, polyvinylpyrrolidone, glyceryl and PEG-100 stearate; Emulsion w/o with polyvinylpyrrolidone, polyquaternium 51, and cetyl dimeticone copolyol	Millerioux et al. [60]
Porcine ear skin	Warfare agent VX	Unprotected membrane	Cream with polyperfluoromethylisopropyl ether (Fomblin^TM^ HC/R) and polytetrafluoroethylene	Chilcott et al. [55]
Human abdominal skin	Nickel nanoparticles	Unprotected membranes; membrane covered with vehicle (without chelating agent)	Commercial skin care product with, e.g., pentetic acid, cetostearyl alcohol, chitosan, paraffin; Commercial cream formulation with, e.g., ceramide 3, palmitamide MEA, cholesterol, squalane, and xanthan gum	Magnano et al. [61]
Human abdominal skin	Nickel powder	Unprotected membranes	Commercial skin care product with, e.g., pentetic acid, cetostearyl alcohol, chitosan, paraffin; Commercial cream formulation with, e.g., ceramide 3, palmitamide MEA, cholesterol, squalane, and xanthan gum	Magnano et al. [62]
3D skin model Episkin™	Caffeine	Membrane covered with petrolatum	Commercial cream products differed in composition and water content (20–70%, *w*/*w*)	Casiraghi et al. [10]

Note: PEG-100—Polyethylene Glycol-100; CMC—carboxymethylcellulose sodium; MEA—monoethanolamine salt.

**Table 4:** General recommendations for barrier efficacy testing with an in vitro penetration approach.

Variables	Recommendations
Membrane-linked variables	
Tissue sample	Human samples from surgical biopsies, cosmetic surgeries, and cadavers Porcine skin with high resemblance to human skin is recommended among animal models Fresh and frozen samples are adequate Freezing and storage procedure should be specified Tissue integrity should be confirmed before and at the end of the test Prolonged conditioning with cosolvents or surfactants should be avoided
Synthetic membrane	Effective for screening studies Initially check for compatibility with tested products and irritant agents Cellulose, polycarbonate, silicone, and lipid-based membranes are acceptable Membrane integrity should be confirmed before and at the end of the test
Process parameters	
Acceptor solution	A simple solution, e.g., buffered saline solution, can be used For freshly excised tissue, a physiological solution (e.g., cell culture medium) should be considered Volume at least three times larger than required to obtain a saturated solution of the chemical agent (sink conditions provided) The presence of solubility-enhancing agents should be justified Non-physiological pH values are acceptable depending on the chemical agent’s solubility Strongly hypotonic or hypertonic media should be used cautiously
Model chemical agent	Chemicals characterized by high permeability are recommended Both hydrophilic and lipophilic substances are acceptable An infinite dose in the donor chamber to assure the steady-state flux Pretreatment time with the chemical agent should be specified
Data presentation	Data expressed as a percentage of protection relative to the control
Control studies	The barrier effect compared with the reference product with proven barrier efficacy, if relevant
Instrumental variables	
Measuring system	Franz diffusion, in-line, side-by-side cells are acceptable The chamber should be warmed up to skin temperature
Analysis time and sampling schedule	As short as possible to avoid impairment in tissue integrity over time Sampling schedule should distinguish the time point necessary to disrupt barrier efficacy

## Data Availability

No new data were generated in this review. The findings are based on a synthesis of existing literature.

## Abbreviations

The following abbreviations are used in this manuscript:

AEA	N-acetylethanolamine
AKT	protein kinase B
BCS	biopharmaceutic classification system
FLG	filaggrin
HAS3	hyaluronan synthase 3
HMOX1	heme oxygenase-1
IVL	involucrin
LOR	loricrin
NF-κB	nuclear factor kappa-light-chain-enhancer of activated B cells
PEA	N-palmitoylethanolamine
PEG	polyethylene glycol
POX	paraoxon
PTFE	polytetrafluoroethylene
SCH	stratum corneum hydration
SLS	sodium lauryl sulfate
Src	proto-oncogene tyrosine protein kinase
TEWL	transepidermal water loss
